# Outcomes of cardiac surgery in Jehovah’s Witness patients: A review

**DOI:** 10.1177/0267659120980375

**Published:** 2020-12-16

**Authors:** Aimee-Louise Chambault, Louise J Brown, Sophie Mellor, Amer Harky

**Affiliations:** 1 Medical School, College of Medical and Dental Sciences, University of Birmingham, Birmingham, UK; 2 Department of Cardiothoracic Surgery, Liverpool Heart and Chest, Liverpool, UK; 3 Department of Integrative Biology, Faculty of Life Sciences, University of Liverpool, Liverpool, UK; 4 Liverpool Centre for Cardiovascular Science, University of Liverpool and Liverpool Heart and Chest Hospital, Liverpool, UK; 5 Department of Cardiac Surgery, Alder Hey Children Hospital, Liverpool, UK

**Keywords:** Jehovah’s Witness, bloodless surgery, transfusion, outcomes, cardiac surgery, blood products

## Abstract

**Objective:**

To review current literature evidence on outcomes of cardiac surgery in Jehovah’s Witness patients.

**Methods:**

A comprehensive electronic literature search was done from 2010 to 20th August 2020 identifying articles that discussed optimisation/outcomes of cardiac surgery in Jehovah’s Witness either as a solo cohort or as comparative to non-Jehovah’s Witnesses. No limit was placed on place of publication and the evidence has been summarised in a narrative manner within the manuscript.

**Results:**

The outcomes of cardiac surgery in Jehovah’s Witness patients has been described, and also compared, to non-Witness patients within a number of case reports, case series and comparative cohort studies. Many of these studies note no significant differences between outcomes of the two groups for a number of variables, including mortality. Pre-, intra and post-operative optimisation of the patients by a multidisciplinary team is important to achieve good outcomes.

**Conclusion:**

The use of a bloodless protocol for Jehovah’s Witnesses does not appear to significantly impact upon clinical outcomes when compared to non-Witness patients, and it has even been suggested that a bloodless approach could provide advantages to all patients undergoing cardiac surgery. Larger cohorts and research across multiple centres into the long term outcomes of these patients is required.

## Background

According to the latest figures from 2019, there are currently over 8 million Jehovah’s Witnesses (hereafter referred to as Witness/Witnesses) worldwide, spread across 240 countries.^1^ Whilst only equating to 0.1% of the world’s population, this unique sub-set of patients provides a distinctive challenge to medical professionals, due to their firmly held beliefs regarding blood transfusion and the use of blood products.^2,3^ The basic principle of these beliefs is the refusal of both blood transfusion and blood products due to interpretations made from the Bible, Genesis 9:4 and Acts 15:28–29.^2,4^ The abridged quote from the Acts reads that individuals must ‘abstain. . .from blood’, and is just one quote which helps to formulate Witnesses stance on blood transfusions.^2,5^ These interpretations of the Bible were first formulated by a group of Bible students in Pennsylvania, and through the dissemination of their work, the Witness following has grown substantially.^1,6^ The community now has its own governing body, as well as ‘The Watchtower Bible and Tract Society’, which aims to promote the societies views through use of education.^7–10^ Further to this, within the UK there are Hospital Liaison Committees for Jehovah’s Witnesses, which can provide support and advice to medical professionals.^11^ This is particularly important for clinicians to consider, as prospective Witness patients present a complicated array of social, legal and ethical challenges.^12^ This is especially true within the field of cardiac surgery and hence needs careful consideration.

**Figure 1.**
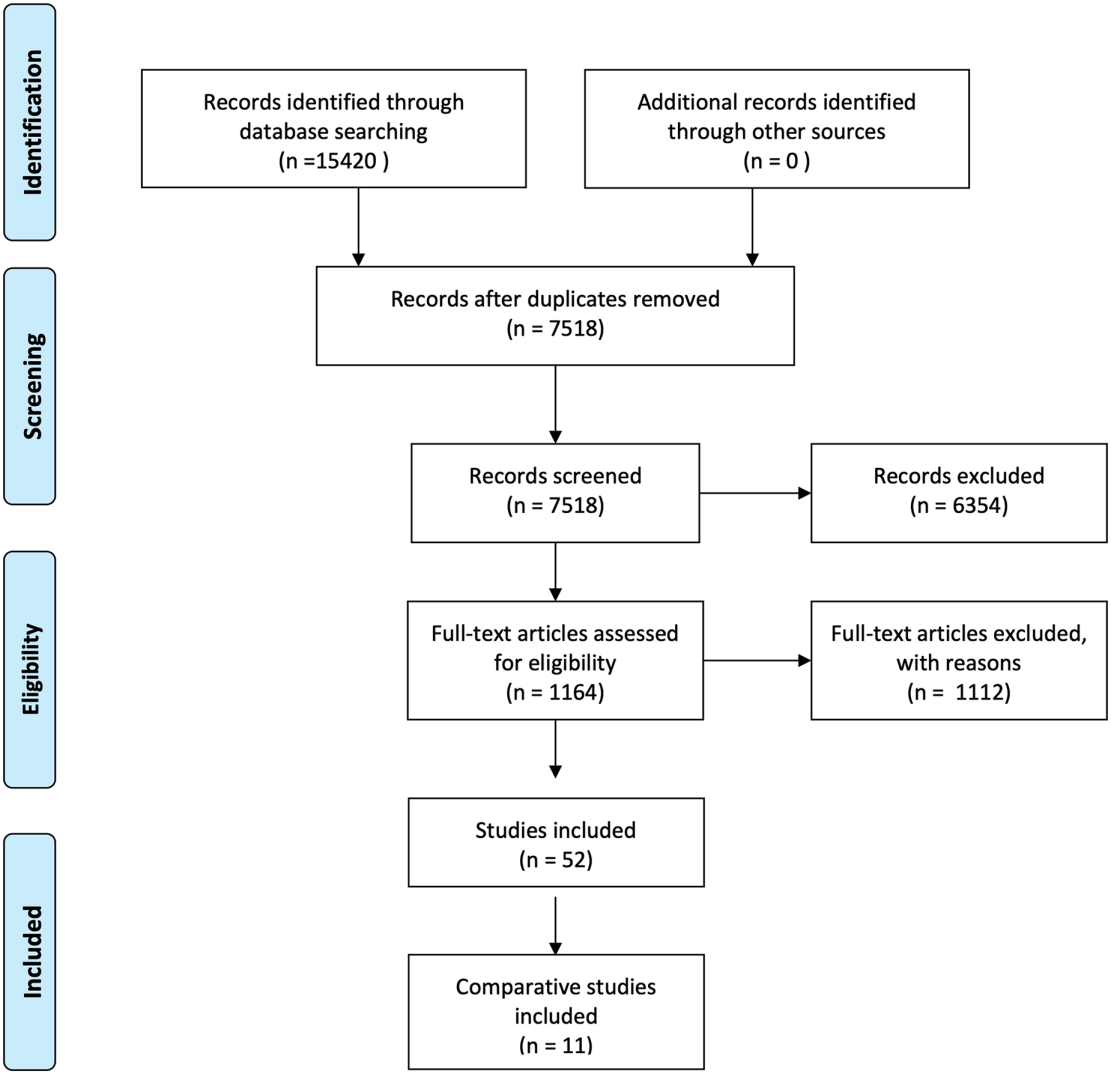
Number of studies screened and included (based on the PRISMA flow diagram).^13^

A comprehensive literature search was performed between the years 2010 and 2020 in PubMed, Scopus, Google Scholar, and Embase. Key search terms included ‘Jehovah’s Witness’, ‘Cardiac surgery’ and corresponding synonyms for outcomes. Non-English papers were excluded. Figure 1 shows the number of studies screened and included. This review examines pre-published data and therefore no human ethics or informed patient consent was required.

## Cardiac surgery and Jehovah’s Witness patients

Witness patients undergoing cardiac surgery is not without risk, primarily due to this surgery carrying an increased association with bleeding and the inability to transfuse blood products. In 1964, open heart surgery using cardiopulmonary bypass (CPB) on Witnesses was first described, made possible by the development of bypass techniques.^14^ Witnesses are often not considered for cardiac surgery due to the increased risk of morbidity and mortality, with early research demonstrating a mortality rate of 7%–10%.^15^ This perceived risk is affected by the population being referred for cardiac surgery; at later stages in life, with multiple comorbidities, and often the use of new anticoagulants.^4^ As research has evolved, there has been a decrease in mortality rates demonstrated by a range of larger studies;^16,17^ whether this is due to improvements in blood management strategy, technology advances, or careful patient selection is unclear. The majority of these findings are limited to adults, however research has now been extended to include infants and children, highlighting similar results and emphasising the need for more liberal, instead of restrictive, transfusion strategy.^18,19^ Greater complications arise when the size of the patient is taken into consideration,^19^ largely due to the resultant increase in haemodilution from CPB prime volume to the circulating blood volume of neonates and infants.^18,20^ However, Olshove et al.^21^ demonstrated that bloodless cardiac surgery is feasible for this population if a comprehensive blood conservation program is adopted. This can be achieved through careful discussion and a thorough understanding of the patients’ beliefs at the pre-operative stage. This is particularly important to comprehend because not all Witness patients hold identical beliefs and the exact level of acceptance of blood product usage lies with each individual, as discussed below.

## Patient beliefs and considerations

The Bible teachings of Charles Taze Russell are the basis of the beliefs held by Witnesses, which state the transfusion of allogenic whole blood and primary blood components (platelets, white cells, plasma or packed red cells) are unacceptable.^22,23^ Witnesses also decline autologous transfusion, as blood is considered unclean once it has left the body, and blood sampling for use in cross-matching.^23^ The use of a number of related treatments is a matter of personal decision for the individual. The derivatives of primary blood products include albumin, coagulation factors, interferons, haemoglobin and globulins/immunoglobulins. Autologous procedures, such as haemodilution, cardiac bypass and cell salvage, may also be deemed acceptable. Bloodless extracorporeal membrane oxygenation (ECMO) has also previously been utilised in an adolescent Witness patient.^24^ It is essential to discuss alternatives with each Witness patient to assess their position, including their right to refuse treatment, especially in situations that would result in loss of life or limb. Discussions should be clearly documented in the notes. In emergency situations, most Witnesses carry a signed and witnessed advance decision card to express their wishes in emergencies, refusing allogenic whole blood and blood components, and also autologous pre-donation. If no such card is present, and the patient is known to be a Witness, every effort should be made to avoid the use of blood and blood products, but the ultimate decision rests with the clinician responsible for that patient.^23^ It is vital the clinician is adequately educated regarding the legal and ethical challenges which a Witness case presents in order to optimise patient management.

## Legal and ethical challenges

The key ethical principles which underpin medical practice are non-maleficence, beneficence, autonomy and justice and their utilisation applies to care of any patient undergoing cardiac surgery, including Witnesses.^11,12,23,25^ This is outlined, along with core legal principles, within existing clinical guidelines, as well as case reports, with one notable example by Papalexopoulou et al.^12^ This case helps to demonstrate the ethical principles of beneficence and non-maleficence, as it presents a complex case in which the risk-to-benefit of treatment was vital to assess.^12^ It discusses that it can be deemed inappropriate for clinicians to refuse to treat a patient based on such factors as high risk.^12^ A clinicians choice to treat Witness patients is further noted in guidance by RCS England.^23^ Doctors do have a choice about whether to treat patients who refuse blood transfusion, as the clinicians may deem this a conflict to their role as a doctor, especially in life-saving situations. If so, the refusing clinician must refer the patient to a doctor who is prepared to perform treatment with knowledge of the patients’ views. This must be recorded adequately to prevent claims of misconduct.^23^ Furthermore, the concept of adequate note-taking and record keeping is also considered within guidelines with regards to discussions about treatment options and eventual decision making with patients, allowing for autonomy.^10,23^ Patients with mental capacity have the right to refuse treatment, and this is upheld in both ethical and legal respects, making administration of blood products against the patients will potentially unlawful.^23^ It is imperative that alternative treatments are made available to Witness patients, with a number having been developed over the years.

## Alternatives to blood transfusion

The use of alternative treatments to blood transfusion is widely discussed within the clinical literature. It is especially noted within a number of case reports and case series specific to Witness patients. One such example is a case report by Robblee et al.,^26^ which describes the novel use of prothrombin complex concentrate and cryoprecipitate in a Witness patient undergoing a redo aortic valve replacement and bypass graft. Additionally, many authors within the literature offer protocols from their own centres for the conservation of blood products and management of patients who refuse these pre, post and peri-operatively.^17,27,28^

In addition to these protocols, guidelines and recommendations exist, produced by the National Institute of Health and Clinical Excellence (NICE), the Royal College of Surgeons of England and the Joint United Kingdom (UK) Blood Transfusion and Tissue Transplantation Services Professional Advisory Committee.^11,23,29^ These provide further clarification on the alternatives to blood transfusions and the key information from these has been summarised in Table 1.

**Table 1.** Alternatives to blood transfusion.

Category	Therapies	Utilisation in cardiac surgery	Suitability for witness patients
Pharmacological	• Tranexamic acid • Recombinant clotting factors • Desmopressin • Erythropoietin • Iron (intravenous & oral) • Thrombopoietin mimetics • Aprotinin • Tissue sealants	Aprotinin was utilised in cardiac surgery before temporary withdrawal in 2008, and is now used in patients where high bleeding risk outweighs drug side effects, which include renal failure. Recombinant activated Factor VII is typically used to treat bleeding when Haemophilia A or B patients undergo surgery, but is also utilised off-label within cardiac surgery	Generally accepted but dependent on the individuals personal beliefs
Autologous transfusion	• Cell salvage: intraoperative and postoperative • Predeposit autologous donation • Normovolaemic haemodilution	Intraoperative cell salvage with tranexamic acid is recommended for use in cardiac surgery by NICE, due to high anticipated blood loss. Acute normovolaemic haemodilution is commonly utilised in cardiac bypass surgery but includes risks of cardiac ischaemia and volume overload	Cell salvage and haemodilution can be classed as acceptable. Predeposit autologous donation is generally considered unacceptable
Operative approach and technique	• Consider laparoscopic/endoscopic/transcatheter approaches where applicable • Optimise vasoconstriction using medication, clamps and tourniquets as appropriate • Utilise regional anaesthesia where appropriate • Utilise deliberate, controlled hypotension and hypothermia • Utilise equipment such as harmonic scalpels and radiofrequency ablation techniques	The utilisation of minimal approaches has been advocated in cardiac surgery in recent years. One key example of this is the utilisation of transcatheter device closure as opposed to surgical closure of atrial and ventricular septal defects	Majority of these considerations will be applicable as they do not utilise blood products specifically

Potential therapeutic options available and currently advocated as alternatives to blood transfusions as per current guidelines and recommendations.

Novel therapies are being developed as further alternatives to blood transfusions, such as artificial blood substitutes. These include perfluorochemical-based substitutes, recombinant-Hb and the use of stem cells for the production of red blood cell alternatives. Although not currently approved for use in the UK, these provide an example of on-going developments in the subject area.^30,31^

One key factor, which is referenced frequently throughout the clinical literature and within guideline criteria, is the importance of discussion of various therapeutic options with the patient.^10,11,23^ This is in order to establish which therapy is most suitable for the patient, however, evidence of direct comparisons between therapies in Witness patients appears to be lacking. One such example of a comparison comes from a study comparing anti-fibrinolytics in 59 Witness patients (aprotinin, TXA and no anti-fibrinolytic use), which found that aprotinin reduced median drain output compared to TXA or no agent used (330 vs 500 vs 440 mL, respectively), but that the agent used made no difference to mortality, morbidity or LOS.^32^ However, the study stated that due to possible bias within the selection of patients, these results were not fully conclusive.^31^ Aprotinin was removed from the market in 2008 and is now only utilised in those with heavy bleeding.^11,33^

Overall, clinicians must establish the level of acceptable use of blood products with the patient pre-operatively and additionally discuss the risks of lack of use in emergency situations.^5,10,11,23^ Furthermore, the utilisation of a multidisciplinary team (MDT) approach is highlighted frequently within the literature as being central to the establishment of a bloodless protocol for Witness patients.^34–36^ This is especially important given that blood transfusions themselves have associated risks and consequences, and that in some circumstances a bloodless regime may in fact be a good treatment approach.^10,37^

## Blood transfusions and associated risks

Blood transfusions are not without risk, for example acute transfusion reactions, ranging from mild to life-threatening, such as mild urticaria, acute haemolytic reactions, transfusion-associated circulatory overload and transfusion-related acute lung injury.^38^ In several cases, the risk is greater when transfusing blood products versus management without transfusion. Engoren et al.^39^ found that even after comorbidities, age and additional confounding variables were accounted for, the 5-year mortality rate had increased by 70% in patients that received a blood transfusion compared to those who did not. Similarly, research including 10,289 patients receiving blood transfusions demonstrated, after controlling factors such as demographics and comorbidities, a reduction, not only in immediate, but in long-term survival.^40^

## Optimisation of outcomes

### Pre-operative

It is important for both clinicians and service providers to assess whether they are adequately equipped to provide Witnesses patients with the best care.^12^ A number of complex cardiac surgery case reports demonstrate this and express the importance of referral to tertiary centres.^12,28^ This was especially noted in a case report by Papalexopoulou et al.,^12^ where a Witness patient was eventually operated on for an aortic dissection following previous assessment at two other centres.

A further factor that could be assessed at the pre-operative stage for Witness patients is the relative risk of patients requiring a blood transfusion during surgery. A number of tools have been developed to aid with this, such as the use of the ‘Transfusion Risk And Clinical Knowledge (TRACK) score’, which was utilised by Kim et al.^41^ for evaluating Witness patients, as well as the use of the ‘Transfusion Risk Understanding Scoring Tool (TRUST)’ utilised on Witness patients by Moraca et al.^4^

There are a number of other standard pre-operative steps which clinicians must undertake in order to appropriately manage Witness patients. One of the most important and frequently studied in the literature is the optimisation of pre-operative haemoglobin levels through the use of Erythropoietin (EPO) and either oral or IV iron.^3,27,42–44^ The use of EPO in particular was explored by Duce et al.^8^ in a matched cohort study, which compared patients who were treated with EPO and declined blood transfusion, to controls who did not receive EPO at all. The study noted that there were no clinically significant differences in outcomes measured between the two cohorts, demonstrating the positive impact of EPO for patients refusing transfusion and hence supports its use for Witness patients.^8^

Eleven comparative studies discussing outcomes between Witnesses and non-Witnesses were found^2,16,21,45–52^ (Table 2) and these often gave agents to increase the preoperative Hb. This varied between studies and makes comparison more challenging. Six of the studies reported preoperative Hb levels,^45–48,51,52^ with three of these showing higher levels for the Witness group (Witness vs non-Witness: 13.7 vs 12.8 g/dL, *p* = 0.01;^45^ 13.9 vs 12.3 g/dL, *p* < 0.0001;^47^ 13.6 vs 12.9 g/dL, *p* = 0.01^48^). Similar preoperative haemoglobin levels were reported in Witness only studies (12.1 ± 1.3,^53^ 13.91,^8^ 14.1^31^ and 14.5 g/dL^54^). Postoperative haemoglobin levels were reported in five of the 11 comparative studies, with three showing a significantly higher result in the Witness group (Witness vs non-Witness: 10.8 vs 9.9 g/dL, *p* = 0.003;^45^ 11.7 vs 9.8 g/dL, *p* < 0.0001;^47^ 11 vs 10 g/dL, *p* = 0.003^48^). A non-comparative study reported a similar postoperative haemoglobin level in a Witness population (10.1 ± 1.5 g/dL).^53^ A study in Witnesses undergoing non-cardiac surgery found an increased risk of morbidity and mortality when haemoglobin levels were below 8 g/dL,^55^ with another study reporting similar outcomes within cardiac surgery,^56^ highlighting the importance of increasing Hb levels preoperatively.

**Table 2.** Outcomes from identified studies comparing Jehovah’s Witness and non-Jehovah’s Witness patients undergoing cardiac surgery (*indicates significant difference (*p* < 0.05) between JW and control groups).

Author	Year	Study period	Group	*N*	Mean age ± SD (years)	Preoperative Hb (g/dL)	CPB time (min)	Cross clamp time (min)	Post-operative Hb (g/dL)	In hospital mortality	30-Day mortality	Hospital LOS (days)	ICU LOS (h)	Mechanical ventilation	Post-operative blood loss (mL)	Reop for bleeding	AKI	Stroke	MI	Infection
DeCoste et al.^46^	2018	01/2008–06/2013	JW	25	63.4	14	140.2	–	100	2 (8%)	–	10.3	138.8	–	–	–	2 (8%)	–	–	1 (4%)
			Control	25	64.6	13.3	118.5	–	95	0	–	13.4* (*p* = 0032)	140.8	–	–	–	5 (20%)	–	–	4 (16%)
Valle et al.^51^	2017	2008–2016	JW	16	60.6 ± 12.1	13.6	58.5	38.5	9.2	3 (18.8%)	–	6.5	–	>48 h: 4 (25%)	–	1 (6.3%)	3 (20%)	0	1 (6.3%)	–
			Control	48	63.3 ± 11.1	12.7	67.5	48.0	9.4	2 (4.2%)	–	7.0	–	>48 h: 4 (8.3%)	–	3 (6.3%)	1 (2.1%)* (*p* = 0.039)	2 (4.2%)	2 (4.2%)	–
Willcox et al.^52^	2020	2007–2018	JW	118	68	13.5	83	–	–	3%	–	6	–	>48 h: 4%	4 hrs post op: 150	4%	8%	2%	1%	–
			Control	118	68	13.2	80	–	–	2%	–	7	–	>48 h: 5%	4 h post op: 175	5%	22%* (*p* = 0.003)	2%	3%	–
Bhaskar et al.^45^	2010	2002–2005	JW	49	65.3	13.7	110.3	–	10.8	-	6 (3.06%)	–	24.1	14 h (6.1%)	24 h: 466.8	10 (5.05%)	6 (3.06%)	7 (3.6%)	2 (1.02)	–
			Control	196	61.2	12.8* (*p* = 0.01)	111.8	–	9.9* (*p* = 0.003)	-	1 (2.04)	–	28.4	12 h (8.8%)	24 h: 843* (*p* = 0.0001)	1 (2.04)	1 (2.04%)	1 (2.04%)	0	–
Olshove et al.^21^	2017	–	JW	5	7.6 ± 9.4	–	76 ± 20	41 ± 29	–	0	–	3.4 ± 1.8	–	–	mL/kg/h: 0.8 ± 0.6	–	0	–	–	0
			Control	10	7.4 ± 14.2	–	95 ± 34	58 ± 30	–	0	–	3.9 ± 1.4	–	–	mL/kg/h: 0.7 ± 0.9	–	0	–	–	0
Guinn et al.^47^	2015	2005–2012	JW	45	66	13.9	–	79.2	11.7	0	0	9	–	–	–	–	73.3%	–	–	0
			Control	90	64	12.3* (*p* < 0.0001)	–	65.2	9.8* (*p* < 0.0001)	0	0	7	–	–	–	–	77.8%	–	–	7.8%
Pattakos et al.^16^	2012	1983–2011	JW	322	62 ± 15	–	–	–	–	10 (3.1%)	–	7.0	25	–	–	12 (3.7%)	2 (0.2%)	7 (2.2%)	1 (0.3%)	0
			Control	48986	62 ± 14	–	–	–	–	2216 (4.5%)	–	8.0* (*p* < 0.001)	48* (*p* < 0.001)	–	–	3596* (7.5%) (*p* = 0.01)	1554* (6.4%) (*p* = 0.002)	1415 (2.9%)	765 (1.6%)	445 (0.9%)
Marinakis et al.^2^	2016	1991–2012	JW	31	62 ± 15	–	104 ± 35	61 ± 27	–	1 (3%)	–	12.9 ± 7.6	103.2 ± 93.6	1.26 ± 2.26 days	209 ± 235	–	–	0	0	1 (3%)
			Control	62	62 ± 15	–	98 ± 36	59 ± 29	–	1 (2%)	–	10.9 ± 6.6	72 ± 33.6	0.89 ± 0.55 days	308 ± 367	–	–	0	1 (2%)	1 (2%)
Vaislic et al.^50^	2003	–	JW	40	69 ± 5	–	59 ± 21	51 ± 15	–	0	0	9	48	2 hrs	312 ± 141	1 (2.5%)	–	0	0	0
			Control	40	71 ± 8	–	62 ± 24	53 ± 16	–	0	0	11	48	2 hrs	721 ± 619* (*p* < 0.05)	1 (2.5%)	–	0	2	0
Reyes et al.^48^	2007	1998–2006	JW	59	62.5	13.6	110.3	77.7	11	4 (6.8%)	–	12.2	50.4	17.1 hrs	446.8	3 (5.1%)	1 (1.7%)	3 (5.1%)	2 (3.4%)	–
			Control	59	62.5	12.9* (*p* = 0.01)	111.8	74.3	10* (*p* = 0.003)	5 (8.5%)	–	17.3* (*p* = 0.05)	88.8	48.5 hrs	813.2	2 (3.4%)	1 (1.7%)	3 (5.1%)	1 (1.7%)	–
Stamou et al.^49^	2006	1990–2004	JW	49	62.7 ± 9.5	–	–	–	–	3 (6%)	–	7.1 ± 8.0	1.5 ± 1.3	>48 h: 2 (4%)	–	3 (6%)	1 (2%)	1 (2%)	0	–
			Control	196	60.3 ± 15.2	–	–	–	–	16 (8%)	–	7.0 ± 5.3	2.0 ± 3.1	>48 h: 8 (4%)	–	16 (8%)	7 (4%)	8 (4%)	1 (1%)	–

AKI, Acute Kidney Injury; CPB, Cardiopulmonary Bypass; Hb, Haemoglobin; ICU, Intensive Care Unit; JW, Jehovah’s Witness; LOS, Length of Stay; MI, Myocardial infarction; SD, Standard Deviation.

### Intra-operative

A number of intra-operative management techniques were noted previously in this paper in the section: Alternatives to Blood Transfusion. The predominant technique evident within the literature is intraoperative cell salvage due to its general acceptability with Witness patients.^11,12,52,57^ This is in addition to the use of intraoperative normovolaemic haemodilution, which was utilised in 37 of the 45 Witness patients undergoing cardiac surgery in a study by McCartney et al.^3^

The utilisation of antifibrinolytics, heparinisation and carefully considered surgical techniques are also discussed within the clinical literature.^3,11,58^ An example of this is the use of sternum bone wax as standard for Witness patients in a study by Emmert et al.^59^ Other operative aspects such as CPB and cross clamp times were not significantly different between Witness and non-Witness groups in various comparative studies (Table 2).

Another point noted from the literature is the importance of distinctions between adult and paediatric patients. This is highlighted within case reports and series, such as that by Boettcher et al.,^60^ who reported on three Jehovah’s Witness infants undergoing cardiac surgery. In this report, these patients were managed using a miniaturised extracorporeal circuit and increased crystalloid cardioplegia.^60^

### Post-operative

Post-operative measures for Witness patients consists of standard post-operative cardiac care with additional considerations. From the literature, avoidance of hypertension and the maintenance of normothermia is key, as is the use of paediatric or low-volume blood bottles for sample collection.^27,53^ Further to this, as can be expected, it is also important to continue the delivery of adjuncts such as IV iron and desmopressin as necessary to patients.^27,53^ Tanaka et al.^27^ additionally suggests that Witness patients can be given haemodynamic support for longer in order to maximise systemic oxygen delivery. Mechanical ventilation is one such example of these measures, however, it has been noted in the literature that this is not a measure undertaken by all clinicians, as no difference was found in ventilation times in seven comparative studies between Witnesses vs controls (Table 2).^2,45,48–52^

Six of the 11 comparative studies in Table 2 which reported the outcome of blood loss did so differently, making direct comparison difficult.^2,21,48,50–52^ However, within the studies themselves, two found that the Witness group experienced significantly less postoperative blood loss compared to the non-Witness control (Witness vs non-Witness: 466.8 vs 843 mL, *p* = 0.0001;^45^ 312 ± 141 vs 721 ± 619 mL, *p* < 0.05^50^). This may be explained by more careful surgery and the blood conserving strategies discussed in the studies. Binder et al.^43^ investigated different transcatheter aortic valve replacement (TAVR) techniques and concluded the use of transfemoral TAVR decreased blood loss when no transfusions were given. Taken together, the results suggest that Witnesses do not have more postoperative blood loss than non-Witnesses, and may even have better outcomes due to strategies employed.

## Emergency situations

It is now considered relatively common to carry out uncomplicated elective cardiac surgery on Witnesses, as this enables meticulous planning and execution of blood management strategies. However, emergency cardiac surgery does not allow for careful planning, presenting a greater challenge and thus increasing the risk of bleeding both peri- and post- operatively. Under such circumstances the doctor has a legal right to determine whether to proceed with a blood transfusion if they cannot clearly ascertain the refusal of blood products.^5^ Emergency cardiac surgery in Witnesses presents additional problems; presentation with severe anaemia and bleeding is inherently difficult to manage without the transfusion of blood. Viele and Weiskopf^61^ found that in Witnesses who did not receive transfusions, and had a haemoglobin of ⩽8 g/dL or a haematocrit of ⩽24%, there was a 37% mortality rate (50 of 134). This has since been supported by Hogervorst et al. who highlighted an association between low haemoglobin levels (<8 g/dL) and levels of mortality and morbidity.^56^ Contrary to this, a study carried out over a 10-year period on 91 Witnesses undergoing cardiac surgery found no significant difference in the risk of mortality or major complications between those who received emergency compared to elective surgery.^53^

It is important to note that differences in outcomes may arise during surgical complications where large amounts of blood are lost before the complication is resolved. This type of event is rare and so may not show in any study. This situation may become non-recoverable for Witnesses, as the blood lost in unable to be replaced, or replaced adequately with alternatives if permitted.

## Jehovah’s Witnesses compared to non-Jehovah’s Witnesses: Outcomes in cardiac surgery

As mentioned previously, 11 studies were identified which compared outcomes for Witnesses and non-Witnesses following cardiac surgery. Table 2 details the outcomes for the comparative studies reviewed. Many other studies were identified that discussed the outcomes of cardiac surgery for Witnesses only, which will be discussed below.

### Mortality

In-hospital mortality figures were given for 10 out of the 11 comparative studies found.^2,16,21,45–52^ None stated a significant difference in mortality rates between Witnesses and non-Witnesses. Witness in-hospital mortality rate in the comparative studies varied between 0% and 18.8%. These figures are similar to those found in non-comparative studies, which ranged from 0% to 14.3%.^8,17,32,44,53,54,58^ One study by Ramiaramanana et al.^54^ split the 153 Witness study population into low and high risk patients using their co-morbidities. Of the 13 classed as high risk, 3 died (23%), which was significantly higher than those in the low risk group (3 of 140 (2.1%), *p* = 0.001).^54^ Jassar et al.^53^ compared mortality rates in Witnesses undergoing elective versus urgent surgery, showing the mortality rate was higher in the elective surgery group (5 vs 0, respectively), although the difference was not significant (*p* = 1.0). This study suggests urgent surgery can be carried out on Witnesses with low risk, despite no preoptimisation of Hb levels.

Thirty-day mortality was reported in a number of studies. Only three of the 11 comparative studies reported the data, but of the three studies, no difference in rates was found.^45,47,50^ Again, non-comparative studies found similar results (comparative: 0%–3.06%;^45,47,50^ non-comparative 0%–5%^4,17,44,58^). Vaislic et al.^17^ reported a drop in 30-day mortality with time, stating 3% in those operated on between 1991 and 2003 and 1% for those between 2003 and 2012. They account for this by better techniques and optimisation preoperatively. A study by Ott and Cooley in 1977 found a 30-day mortality rate of 9.4%,^62^ but this study was more than 40 years ago and also did not distinguish between outcomes for adults and children, which may account for the higher percentage seen.

Longer term survival rates were reported in few studies, with only one of the comparative studies sharing this outcome. Pattakos et al. reported survival estimates of Witnesses were 86%, 69%, 51% and 34% at 5, 10, 15 and 20 years after surgery, respectively, vs 74%, 53%, 35% and 23% for non-Witnesses, showing Witnesses had better survival rates.^16^ Jassar et al.^53^ stated the 1- and 5-year survival rates for Witnesses after cardiac surgery to be 87.3% ± 3.4 and 76.1% ± 5.4, respectively. The 5-year survival rates reported for those undergoing CABG only were reported to be 90.3%.

### Morbidity

All studies found reported a different set of outcomes related to morbidity, making comparison for all stated difficult. The most commonly reported morbidity outcomes for the comparative studies can be found in Table 2 and are discussed below. Overall, there appears to be no increased risk of morbidity among Witness patients.

#### Acute myocardial infarction and stroke

Eight out of 11 comparative studies reported rates of myocardial infarction (MI) postoperatively. None of the studies found a difference in rates between the Witness and non-Witness groups, with results ranging from 0% to 6.3% and 0% to 4.2%, respectively.^2,16,45,48–52^ The rates of MI in studies that included Witnesses only were similar, 0%^8^ and 3%.^58^

Eight of the comparative studies also reported rates of postoperative stroke. Again, none of the studies found a difference between the two groups, with frequency ranging from 0% to 5.1% for both groups.^2,16,45,48–52^ Non-comparative studies found low rates of stroke also (0^4^, 1.1%^53^ and 3%^58^).

#### Acute kidney injury

Acute kidney injury (AKI) was mentioned as an outcome in nine out of the 11 comparative studies. The results are mixed, with several stating no difference in the rates of AKI seen between the two groups,^2,21,45–50^ one study reporting a significant decrease in AKI in the non-Witness group^51^ and two studies showing a significant decrease in AKI for Witnesses.^16,52^ The range of patients with postoperative AKI in the comparative studies was large (0%–73.3% for Witnesses and 0%–77.8% for the non-Witness group). Two of the non-comparative studies found varying rates of AKI among Witnesses, with one study reporting it in 2.5% of patients,^4^ whereas Duce et al.,^8^ which compared outcomes for Witnesses given EPO pre operatively to those not given EPO, found AKI rates to be 47.17% and 41.51%, respectively. The wide difference in rates reported may be accounted for by the varying definition of AKI used and the vast array in type of surgeries carried out in different studies, which makes it problematic to form any definitive conclusion concerning the risk of AKI in Witnesses following cardiac surgery.

#### Infection

Infection rates were not significantly different between the Witness and non-Witness groups in any of the six studies which reported this outcome.^2,16,21,46,47,50^ Witness only studies reported varying infection rates of 0,^4^ 1.1%,^53^ 3%^58^ and 9.43%.^8^ Sepsis was recorded for 2.2% of patients in one study.^53^

#### Length of stay

Overall hospital length of stay (LOS) was reported for 10 out of 11 comparative studies,^2,16,21,46,47,50–52^ with three reporting a significantly decreased LOS for Witness patients compared to the non-Witness group (10.3 vs 13.4 days, *p* = 0.032;^45^ 7.0 vs 8.0 days, *p* < 0.001^16^ and 12.2 vs 17.3 days, *p* = 0.05,^48^ respectively). Studies which only described outcomes for Witnesses stated similar LOS times ranging from 5,^8^ 8.1 ± 4.8,^4^ 9.83 ± 7.92^53^ and 12.81 ± 6.58^44^ days.

Seven of the 11 studies mentioned ICU LOS times,^2,16,45,46,48–50^ with one study finding a difference was between the two groups (Witness vs non-Witness: 25 vs 48 h, *p* < 0.001^16^). Non-comparative studies were found to state a wide range of ICU LOS times, ranging from 24 to 103.68 ± 92.64 h.^4,44,58^ Duce et al.^8^ found that Witnesses not given EPO preoperatively were less likely to remain in ICU for more than 24 h and Ramiaramanana et al.^54^ saw a significant difference in LOS for Witnesses classified as low vs high risk (68.4 vs 156 h, respectively (*p* = 0.01)). The difference in results may be again due to the vast array of different surgeries and techniques used in the studies.

#### Reoperation for bleeding

One comparative study found that Witnesses were less likely to need reoperation for bleeding than the control group (3.7% vs 7.5%, *p* = 0.01, respectively^16^). The other 6 studies that compared the two groups found no difference in this outcome.^45,48–52^ Five non-comparative studies found the following percentages of patients requiring reoperation for bleeding: 0,^44^ 2.2,^53^ 5,^4^ 10^58^ and 11.4%.^54^ Again, the different surgeries in the studies may account for the differences, but the results suggest a low risk of reoperation for bleeding in the Witness population.

#### Quality of life

One study reviewed compared quality of life outcomes following cardiac surgery between 14 Witnesses and 18 non-Witness controls. The authors employed the MacNew questionnaire, which showed no difference in physical (*p* = 0.54), emotional (*p* = 0.12), social (*p* = 0.21) and global (*p* = 0.25) scores between the two populations.^63^

## Limitations and future research

There are several predominant limitations across the studies discussed in this review. Firstly, retrospective studies populate this area of research and are prone to selection bias as higher-risk Witnesses are more likely to be excluded, therefore studies will not accurately reflect all those in need of surgery. Additionally, a large proportion of the study designs are single-centre and nonrandomised.^3,8,57^ There is an increased likelihood that surgeons will be more cautious when operating on Witnesses, this in turn could result in reduced blood loss, which is impossible to measure and thus cannot be accounted for.^56^ However, it is important to note that these research limitations were taken into account and propensity matching employed in some studies, to define the control group and minimise such limitations.^15,57^ Nonetheless, this only accounts for variables that were recorded, when in reality there will have been a greater number of confounding variables that could not be included in the propensity matching.

Only one study mentioned cost comparisons for treatment between the two groups and found no significant difference in total costs for Witnesses and their controls.^47^ More research is needed in this area.

Finally, a large proportion of the research discussed in this review consisted of either very small sample sizes^2^ or included case reports and are therefore subject to all recognised limitations, including retrospective limitations aforementioned, generalisability, causality, information and publication bias, overinterpretation or misinterpretation of data. Future research is needed to provide greater clarity on the outcomes of cardiac surgery in Witnesses that refuse blood products. There needs to be an increased focus on multi-institutional studies with a greater number of participants to validate the research to date,^8,63^ along with readmission rates and long term mortality following discharge,^2^ and a more in depth cost analysis of pre-, intra-, and post-operative optimisation of witnesses during cardiac surgery.^8^

## Conclusion

Jehovah’s Witness patients undergoing cardiac surgery present a unique challenge for clinicians. Good communication with the patient and an MDT approach is required in order to ensure safe and successful treatment. Overall, within the clinical literature, the use of a bloodless protocol for these patients does not appear to significantly impact upon clinical outcomes when compared to non-Witness patients, and it has even been suggested that a bloodless approach could provide advantages to all patients undergoing cardiac surgery. However, further research into the long term outcomes of these patients, especially in larger cohorts and across multiple centres, is still required.
